# Pharmaceutical Waste Management and Awareness at Household Level: A Cross‐Sectional Survey of Katlehong and Lambton in Ekurhuleni Municipality

**DOI:** 10.1002/hsr2.72858

**Published:** 2026-07-25

**Authors:** Sithembile Mduduzi Tshomela, Isaac Tebogo Rampedi, Phyllis Rumbidzai Kwenda

**Affiliations:** ^1^ Department of Geography, Environmental Management & Energy Studies University of Johannesburg Auckland Park Johannesburg South Africa

**Keywords:** awareness, disposal practices, household pharmaceutical waste, storage practices, survey

## Abstract

**Background and Aims:**

With the growing human population, improved access to healthcare, and the increasing burden of disease, the consumption and subsequent disposal of pharmaceuticals is rising. This study assessed and compared household pharmaceutical waste (HPW) storage and disposal, and environmental awareness among residents of Katlehong Township and Lambton Suburb within the Ekurhuleni Metropolitan Municipality.

**Methods:**

A stratified random sampling technique was used to identify and administer a semi‐structured questionnaire to 303 respondents (Katlehong = 221; Lambton = 82). Microsoft Excel and IBM SPSS ver.30 were used to generate descriptive statistics and *χ* ^2^ tests.

**Results:**

The results revealed limited knowledge of what pharmaceutical waste is in Katlehong (39.4%, *n* = 87) and Lambton (42.7%, *n* = 35). Despite this, 77% (*n* = 233) of respondents expressed willingness to adopt proper disposal practices if adequate education and disposal alternatives are provided. Concerning storage, the most frequently adopted method in Katlehong (43.0%, *n* = 95) and Lambton (53.7%, *n* = 44) was inside a medicine box. Residents of Katlehong (61.5%, *n* = 136) and Lambton (72.0%, *n* = 59) mostly threw away HPW together with household general waste. Generally, higher‑income households (> R300,000/year) significantly adopted safe HPW disposal methods (*χ* ^2^ (5, 303) = 20.989, *p* < 0.001, *Φ* = 0.118, RR_(<R500,000/year)_ = 0.245 (95% CI: 0.934–24.341)), when compared to lower‐income households (< R500,000/year). Moreso, regardless of comparatively lower socio‐economic conditions prevailing in the township of Katlehong, the data revealed statistically significant safer pharmaceutical storage practices (*χ* ^2^ (5, 303) = 20.989, *p* < 0.001, *Φ* = 0.121, RR(Kahlehong) = 0.442 (95% CI: 0.204–0.957), as compared to Lambton.

**Conclusion:**

Overall findings revealed poor pharmaceutical storage and HPW disposal, as well as limited awareness in both study areas. However, annual household income and the socio‐economic status of the suburb did significantly influence HPW disposal and pharmaceutical storage practices, respectively. This highlights the urgent need for public education and accessible take‐back systems to promote safe HPW management across both socioeconomic communities while also incorporating targeted interventions to effect change.

## 1 Introduction

Globally, there is a rapidly growing interest and research on awareness about the management of medical wastes, improper disposal of unused or expired pharmaceuticals, and their potential negative impacts on environmental quality and human health [1, 2, 3, 4]. With increasing population growth, growing access to health care, and the rising economic burden of human diseases, the utilization and consumption of medications is rising, thus leading to higher quantities of household pharmaceutical waste (HPW) [5]. According to recent estimates, approximately 4.5 trillion doses of pharmaceuticals were consumed worldwide in 2020, thus reflecting both expanded access to healthcare and long‐term reliance on drugs [6]. Between 30% and 90% of these oral medications are ultimately excreted into wastewater as active compounds or metabolites, thereby making excretion the principal source of household pharmaceutical releases [7]. Given the improper disposal of such waste materials and the risks they pose to people and the environment, there is a need for greater awareness regarding their safe disposal and treatment [8, 9, 10].

Pharmaceutical waste (PW) is defined by the World Health Organization as any pharmaceutical products that are expired, no longer needed, or contaminated, including items that contain or have been contaminated by such materials [11, 12]. This includes unused medications, discarded wellness products, and medicines that are no longer required due to a patient's recovery or death [13]. The unused or expired medication (UEM) within households may be referred to as household pharmaceutical waste (HPW) [1]. Poor management of HPW may constrain the achievement of certain UN's Sustainable Development Goals (SDGs); notably SDG 3—Good health and wellbeing, SDG 6—Clean water and sanitation, SDG 12—Responsible consumption and production [6, 14]. These challenges highlight the need for improved education, policy amendments, and effective waste management systems to align local practices with SDGs.

Some frequently applied methods for disposing of HPWs include flushing them in kitchen sinks, or toilet drains, or throwing them into dustbins; although such practices are detrimental to environmental quality and public health [1, 5, 8]. Once discarded, HPWs enter the environment through several pathways [15, 16]. Secondly, it may be washed away through sinks and toilets, thus adding further pollutants to municipal wastewater. Thirdly, when such wastes are mixed with domestic waste, they may end up in the landfills, where they may leach into soil and water sources, thus posing further risks to ecosystems and human wellbeing [2]. Furthermore, traces of pharmaceutical residues from various drugs have been detected in sewage systems, surface water, groundwater, and even seawater, thereby highlighting the widespread nature of this pollution problem [11, 17].

The World Health Organization (WHO) recommends that unused and expired medications should be managed according to standard and proper procedures for safe disposal, especially given their hazardous properties [10, 18]. According to the Food and Drug Administration, unused, leftover, or expired medications should not be disposed of by flushing them down the kitchen or toilet drains [19]. Instead, they should be disposed of through appropriate methods, such as community drug take‐back programs [5]. Guidelines for handling pharmaceutical waste (PWs) from sources like pharmaceutical industries, hospitals, clinics, research laboratories, or households recommend treating solid and semi‐solid PWs through incineration, pyrolysis, encapsulation, inertization, or engineered landfilling, while liquid PWs should be processed at wastewater treatment plants [12, 20].

Although South Africa has a well‐developed legal and regulatory framework for the management of all waste streams, research on levels of awareness amongst healthcare professionals as well as households regarding the proper handling, storage, and disposal of HPWs is limited [9, 21]. The National Environmental Management: Waste Act (Act No. 59 of 2008) mandates municipalities to create Integrated Waste Management Plans [22]. These plans serve as essential tools for local municipalities to effectively plan and manage waste services, thus ensuring they are both adequate and efficient [22, 23]. However, the persistence of knowledge gaps at both household and professional levels suggests that the existence of regulatory frameworks alone is insufficient without complementary efforts in public education and awareness‐raising initiatives. Furthermore, there is limited empirical evidence comparing HPW management practices across different socio‐economic settings within the same municipality, particularly in South Africa. In addition, the specific drivers of improper disposal and the extent to which awareness influences household behavior are not well understood at the community level. Hence, in contributing further to the existing literature, the present study sought to investigate how households store and dispose of pharmaceutical waste, assess their awareness, and identify the challenges preventing proper waste management. The present study assessed and compared household practices, awareness levels, and perceptions regarding HPW management in two settlements with varying socio‐demographic characteristics within the Ekurhuleni Metropolitan Municipality (EMM) of South Africa.

## 2 Study Area and Research Methodology

### 2.1 Description of Study Area

**Figure 1:**
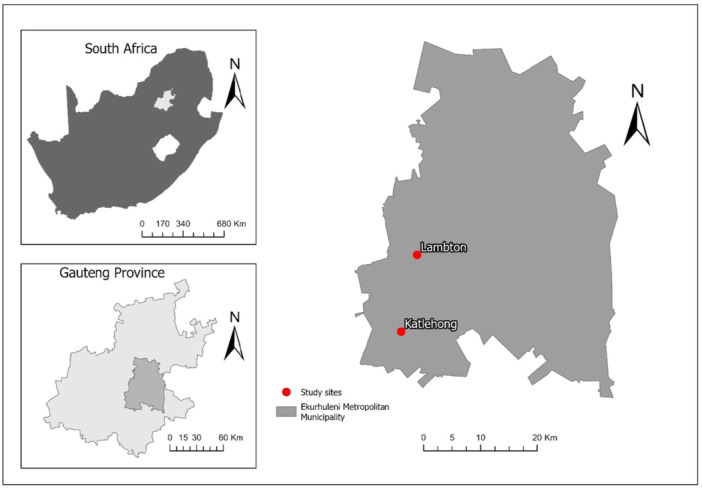
Map showing the location of study areas—Katlehong township and Lambton suburb in Ekurhuleni.

Katlehong township and Lambton suburb in Gauteng province of South Africa were selected as study areas (Figure 1). These two study areas were deliberately selected to represent contrasting socio‐economic settings within the same municipality, with Katlehong reflecting a township context and Lambton representing a suburban context. According to the authors' knowledge, limited to no suburb comparison studies have been done to understand HPW management among Johannesburg residents [24, 25]. Kahlehong and Lambton were chosen because the principal author is familiar with these residential areas, which provided an advantage for navigating the locations for sampling. This allowed for a comparative analysis of HPW management practices under different living conditions while controlling for broader municipal‐level influences.

The City of Ekurhuleni is one of three metropolitan municipalities in Gauteng province, alongside the City of Johannesburg and the City of Tshwane. According to the 2022 Census, Ekurhuleni's population is approximately 4,066,691 people, thus reflecting a significant increase from 3,178,470 in 2011 [26]. The municipality covers an area of 1975 km^2^ and is highly urbanized, with 99.4% of its population living in urban settlements ranging from informal areas to more affluent suburbs [26]. Ekurhuleni faces substantial waste management challenges, exacerbated by its rapidly growing population and industrial activities. Key municipal challenges include inadequate waste collection services, illegal dumping, and limited landfill capacity. For example, according to the Department of Fisheries, Forestry, and Environment (2020), only 66.3% of households receive weekly waste collection services, thereby leaving 33.7% without reliable access, thus rendering the area susceptible to illegal dumping.

Katlehong is a large township situated 35 km east of Johannesburg and south of Germiston, between Thokoza and Vosloorus, along the N3 highway in the EMM (Department of Cooperative Governance and Traditional Affairs, 2020). Together with Thokoza and Vosloorus, it forms part of Kathorus; a collective name derived from the first letters of the three townships. As part of Kathorus, Katlehong forms the second‐largest Black township in South Africa after Soweto. The township faces various socio‐economic challenges, including high unemployment rates, with reports indicating figures around 34.6% [27]. Only about 79% of households have piped water on their property, compared to the municipal average of over 90%, households of about 62% use an upgraded sanitation facility, and about 71% of households are connected to the electrical grid, well below the EMM average of nearly 90% and provincial averages, thus contributing to health risks and social unrest [27].

Lambton is a low‐density, middle‐income suburb of Germiston, the administrative seat of the EMM. According to the 2011 Census, Germiston covers an area of 143.27 km^2^ and has a population of 255,863, thereby resulting in a population density of approximately 1786 people per km^2^ [28]. Furthermore, the area has a total of 91,273 households, with a population density of 637.09 households per square kilometer [28]. In contrast to Katlehong, Lambton has more efficient municipal service delivery, including reliable access to piped water, weekly waste collection, and nearly universal electricity connections [27]. The suburb's residents generally have higher income levels and better access to healthcare and sanitation infrastructure, contributing to improved living standards and reduced environmental health risks compared to surrounding townships [27].

### 2.2 Research Design

#### 2.2.1 Data Collection Techniques

**Figure 2:**
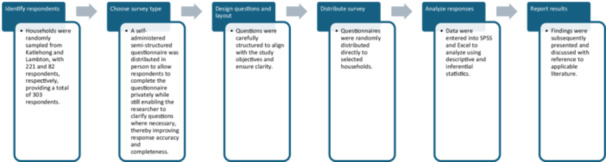
Summary of key steps for the survey research.

This study employed a mixed‐method survey design to gain a better understanding of HPW management practices in Katlehong and Lambton. A semi‐structured questionnaire, which comprised sections on demographic characteristics, storage practices, disposal practices, and perceptions regarding environmental and health risks, was used to gather the primary data. The content validity of the questionnaire was reviewed and advised by a group of experts within the Department of Geography, Environmental Management, and Energy Studies at the University of Johannesburg to ensure the questions were not only easy to understand but were relevant to the objectives of the study. Moreover, to ensure that the questions were clear and understandable, a pilot study of three respondents randomly selected from Kahlehong using the questionnaire was conducted. No amendments were made to the questionnaire before data collection as the questions were clear to the respondents. Data collection was conducted from August 15 to September 20, 2025, during which questionnaires were administered directly to only one member per household who was 18 years or older to ensure consistency, avoid duplication of responses, and adhere to ethical considerations. The six key steps followed in conducting this survey research are illustrated in Figure 2.

#### 2.2.2 Data Analysis and Interpretation

Descriptive and inferential statistics using Microsoft Excel and the Statistical Package for the Social Sciences (IBM SPSS ver. 30) were used to analyze the data. Descriptive statistics, including frequencies and percentages, were used to summarize respondents' demographic characteristics, household pharmaceutical waste (HPW) disposal practices, medicine storage practices, and awareness levels.

To explore the influence of demographic and economic factors on HPW disposal, pharmaceutical storage practices, and awareness across the study areas, *χ* ^2^ tests were conducted. Where statistically significant associations were identified (*p* < 0.05), Phi coefficients (*Φ*) were used to measure the strength of the associations, while Relative Risk (RR) estimates were calculated for relevant binary variables to evaluate the likelihood of specific outcomes between comparison groups.

Additionally, qualitative responses regarding reasons for HPW disposal practices were analyzed using a word‐frequency chat, which was created using a freely available online tool called Word Cloud. The chart was used to identify and visualize the most frequently occurring terms and themes reported by respondents.

#### 2.2.3 Sampling Method

The estimated number of households in Katlehong and Lambton is 467,000 and 2687, respectively [29]. Due to a high refusal rate (*n* = 127, 29.5%), particularly in Katlehong, as well as limited access to respondents in the sparsely populated Lambton suburb, insufficient time allocated for the survey, and resource constraints, the study selected and completed 221 questionnaires from Katlehong and 82 from Lambton. Although the findings cannot be generalized to the entire populations of Katlehong and Lambton, they are sufficient for comparison analysis within the context of the study.

The study employed a stratified random sampling design. Households were stratified by their geographical areas (Katlehong township and Lambton suburb). Stratified random sampling is a probability technique in which the population is divided into relatively homogeneous groups (strata), and then simple random samples are drawn from each group [30]. To preserve randomness, if a selected household was unavailable or lacked an eligible respondent, it was replaced with another randomly selected household rather than following a systematic interval approach. Each stratum was constructed to be exhaustive (covering all eligible households) and mutually exclusive (no overlap between strata).

#### 2.2.4 Ethical Considerations

The study obtained prior informed consent by explaining its objectives, procedures, risks, and respondents' right to withdraw at any time. Thus, everything, including the procedures to obtain the primary data and its analyses, was conducted according to the research ethics permit (Ethics Reference Number: 2025‐07‐10/Tshomela_Rampedi) offered by the University of Johannesburg. Anonymity and confidentiality were ensured; thus, data were securely stored, used only for academic purposes, and handled in compliance with POPIA (2013) without revealing personal identities. Participation was also voluntary, with no penalty for declining or omitting responses. Written or verbal informed consent was obtained from all participants before participation.

## 3 Results and Discussion

### 3.1 Demographic Background of Respondents

Table 1 indicates the demographic profiles of the study areas, combined and separately. Of the 303 respondents, Katlehong accounted for nearly three‐quarters of the total sample (*n* = 221, 72.9%), thus reflecting its higher population density compared to the more affluent and sparsely populated Lambton area (*n* = 82, 27.1%). The gender distribution was almost perfectly balanced, with 151 males (49.8%), 150 females (49.5%), and 2 respondents (0.7%) identified as other gender. In Katlehong, slightly more males (51.6%, *n* = 114) than females (48.0%, *n* = 106) participated, whereas in Lambton, females (53.7%, *n* = 44) were more represented than males (45.1%, *n* = 37).

**Table 1:** Demographic characteristics of respondents and comparisons by residence (Katlehong and Lambton).

Variable	Category	Total *N* (%)	Katlehong *N* (%)	Lambton *N* (%)
Gender	Male	151 (49.8)	114 (51.6)	37 (45.1)
	Female	150 (49.5)	106 (48.0)	44 (53.7)
	Other	2 (0.7)	1 (0.5)	1 (1.2)
Age	20–29	90 (29.7)	65 (29.4)	25 (30.5)
	30–39	89 (29.4)	58 (26.2)	31 (37.8)
	40–49	63 (20.8)	45 (20.4)	18 (22.0)
	50–59	37 (12.2)	32 (14.5)	5 (6.1)
	60–69	20 (6.6)	17 (7.7)	3 (3.7)
	≥ 70	4 (1.3)	4 (1.8)	0 (0.0)
Highest qualification	Some primary school	6 (2.0)	6 (2.7)	0 (0.0)
	Primary school	4 (1.3)	3 (1.4)	1 (1.2)
	Some high school	56 (18.5)	44 (19.9)	12 (14.6)
	Matric	119 (39.3)	84 (38.0)	35 (42.7)
	Post‐matric diploma/certificate	35 (11.6)	27 (12.2)	8 (9.8)
	Bachelor's degree	61 (20.1)	43 (19.5)	18 (22.0)
	Post‐graduate degree (honours, master's, PhD)	22 (7.3)	14 (6.3)	8 (9.8)
Household income	Lower income (R 0–R50,000)	185 (61.1)	131 (59.3)	54 (65.9)
	Emerging middle class (R100,000–300,000)	81 (26.7)	64 (29.0)	17 (20.7)
	Realized middle class (R300,001–500,000)	27 (8.9)	20 (9.0)	7 (8.5)
	Upper middle class (R500,001–750,000)	8 (2.6)	5 (2.3)	3 (3.7)
	Emerging affluent (R750,001–1,000,000)	1 (0.3)	1 (0.5)	0 (0.0)
	Affluent (R1,000,000)	1 (0.3)	0 (0.0)	1 (1.2)
Household type	Private house	119 (39.3)	90 (40.7)	29 (35.4)
	Town house	67 (22.1)	58 (26.2)	9 (11.0)
	Flat/apartment	20 (6.6)	4 (1.8)	16 (19.5)
	Estate	4 (1.3)	4 (1.8)	0 (0.0)
	Rented house	62 (20.5)	39 (17.6)	23 (28.0)
	Retirement village/old age home	1 (0.3)	1 (0.5)	0 (0.0)
	Informal dwelling	22 (7.3)	17 (7.7)	5 (6.1)
	Other	8 (2.6)	8 (3.6)	0 (0.0)
Number of people in a house	1 person	31 (10.2)	13 (5.9)	18 (22.0)
	2 persons	38 (12.5)	25 (11.3)	13 (15.9)
	3 persons	55 (18.2)	37 (16.7)	18 (22.0)
	4 persons	54 (17.8)	40 (18.1)	14 (17.1)
	5 persons	63 (20.8)	50 (22.6)	13 (15.9)
	6 persons	22 (7.3)	17 (7.7)	5 (6.1)
	7 persons	18 (5.9)	18 (8.1)	0 (0.0)
	8 persons	8 (2.6)	8 (3.6)	0 (0.0)
	Over 9 persons	14 (4.6)	13 (5.9)	1 (1.2)

The age profile was relatively youthful, with 59.1% of respondents aged between 20 and 39 years, while only 1.3% were aged 70 or above, thus indicating that most respondents represented the economically active population. Educational attainment varied, but a substantial proportion had at least completed secondary schooling, with 39.3% holding a matric certificate and 20.1% a bachelor's degree. Household income levels reflected a predominantly lower‐income sample, with 61.1% reporting annual incomes below R50,000 (ZAR values; approximately R50,000 ≈ USD2700/£2100, depending on exchange rates). Housing characteristics illustrated diverse living arrangements, with most respondents living in private houses, townhouses, or rented dwellings. Household size was relatively small, with 56% of households containing 3–5 members. Overall, these demographics portray a largely young, lower‐income but relatively educated population, while also making clear the socio‐demographic and economic contrast between Katlehong and Lambton.

#### 3.1.1 Types of Unused or Unwanted Pharmaceuticals

**Figure 3:**
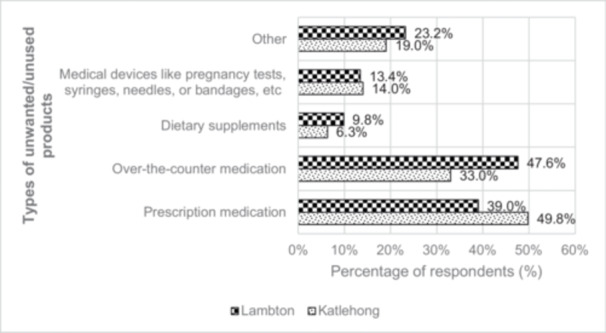
Comparison of unwanted or unused pharmaceutical products reported in households between Katlehong and Lambton (multiple responses).

The kinds of unused/unwanted pharmaceutical or nutraceutical products kept in households were explored in the residential areas sampled. As shown in Figure 3, comparison of unwanted or unused pharmaceutical products reported in households between Katlehong and Lambton (multiple responses), prescription medicines were the most frequently reported, found in nearly half of all households (46.9%), with Katlehong showing a slightly higher prevalence (49.8%) than Lambton (39.0%). Over‐the‐counter (OTC) products followed, mentioned in 37.0% of households, with a marked difference between locations: 47.6% in Lambton compared to 33.0% in Katlehong. These discrepancies suggest that residents in Lambton may be purchasing more non‐prescription items, possibly due to easier access to pharmacies and relatively higher disposable incomes. Similar trends have been reported in studies conducted in urban and higher‐income settings, where OTC medicine use is more prevalent due to accessibility and relatively higher purchasing power [31].

#### 3.1.2 Views on Using Medicines After Expiry Date

**Figure 4:**
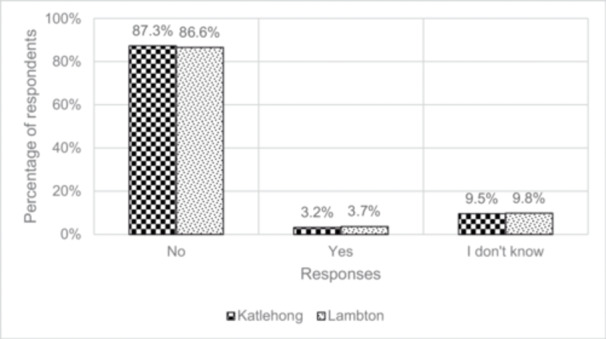
Respondents' views (%) when asked the question “Would you still use your stored medicine if you knew it was expired?” by place of residence.

The study aimed to gather respondents' views on the use of expired medications to assess their awareness of the dangers associated with administering such medicines. Some individuals may not even be aware of the importance of checking expiry dates. The collected opinions were used to evaluate whether expiry dates influence respondents' decisions to discontinue using medications, as existing literature suggests that 29.3% of medicines stored in households are already expired [32, 33, 34]. Figure 4 shows that most respondents in both Katlehong (87.3%) and Lambton (86.6%) reported that they would not use medicines after their expiry date. Only a small proportion, 3.2% in Katlehong and 3.7% in Lambton, said they would use medicines after expiry, while 9.5% and 9.8% respectively were unsure. These findings indicate a shared understanding across the two areas that expired medicines are unsafe, though a small group still displays some uncertainty.

#### 3.1.3 Influence of Demographic and Economic Factors on HPW Disposal

Safe disposal practices were examined across various demographic (gender, age, and place of residence) and economic variables (employment status, education level, and household income). The *χ* ^2^ test assessed whether there were significant associations between these variables and safe disposal practices. As shown in Table 2, the results indicated that there was no statistically significant association between safe disposal practices and gender (*χ* ^2^ (2, 303) = 0.719, *p* = 0.698), age (*χ* ^2^ (5, 303) = 1.548, *p* = 0.907), employment status (*χ* ^2^ (5, 303) = 2.612, *p* = 0.759), education level (*χ* ^2^ (6, 303) = 7.291, *p* = 0.295), or place of residence (*χ* ^2^ (5, 303) = 0.314, *p* = 0.575). This suggests that these demographic factors did not significantly explain how participants disposed of unused or expired medicines.

**Table 2:** *χ* ^2^ test results of safe disposal vs. demographic variables.

Variable	*χ* ^2^ (df)	*p* value
Gender	0.719 (2)	0.698
Age	1.548 (5)	0.907
Employment status	2.612 (5)	0.759
Education level	7.291 (6)	0.295
Household income	20.989 (5)	< 0.001
Place (suburb)	0.314 (1)	0.575

**Figure 5:**
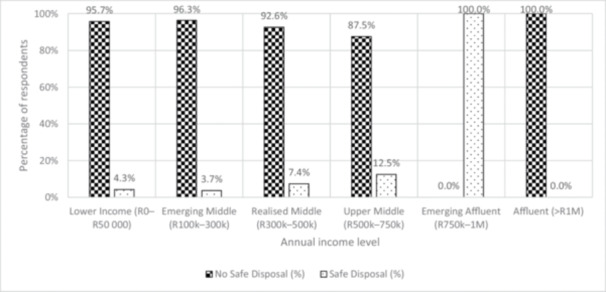
Proportions of safe and unsafe disposal in households of varying incomes.

However, a statistically significant association was found between household income and safe disposal practices (*χ* ^2^ (1, 303) = 20.989, *p *< 0.001). This indicates that income level significantly influenced safe disposal practices. Generally, the majority of participants across all income groups reported unsafe disposal practices. However, relatively higher proportions (≥ 12.5%) of safe disposal were observed among higher‐income categories (≥ R750,001/year) compared to (≤ 7.4%) lower‐income groups (≤ 750,000/year) (Figure 5).

#### 3.1.4 Magnitude and Direction of the Cause‐and‐Effect Relationship Between Household Income and HPW Safe Disposal Practices

To further clarify the size and direction of the relationship as well as the risk associated with the economic variable, household income, on HPW safe disposal practices, the Phi coefficient (*Φ*) and a Relative Risk (RR) were calculated, respectively. As shown in Table 3, unsafe disposal practices were more prevalent among participants from lower‑income households, with 95.5% reporting unsafe disposal compared to 81.8% among higher‑income households.

**Table 3:** Safe disposal practices by household income category (%).

Household income category	Unsafe disposal (%)	Safe disposal (%)	Total (%)
Lower income (≤ R500,000)	95.5	4.5	100
Higher income (> R500,001)	81.8	18.2	100

The strength of the association was positive though weak, as indicated by the Phi coefficient (*Φ* = 0.118). This signified that, in this study, safe disposal increased with a rise in household income. Risk estimate analysis further indicated that respondents from higher‑income households were approximately 4.8 times more likely to report safe disposal practices compared to those from lower‑income households (RR_lower income_ = 0.245). However, the confidence interval around this estimate was wide (95% CI: 0.934–24.341), thereby reflecting uncertainty as a result of the small number of participants in the higher‑income category. Individuals with higher incomes may have better access to information, resources, or facilities that support proper medicine disposal, as previously observed in community‑based household studies from low‑ and middle‑income settings where unsafe disposal practices were strongly linked to limited access to formal take‑back systems [2, 35]. These findings reinforce the importance of considering socio‐demographic and economic context when designing interventions aimed at promoting safe pharmaceutical waste disposal.

#### 3.1.5 Comparison of HPW Disposal Practices Between Study Areas

**Figure 6:**
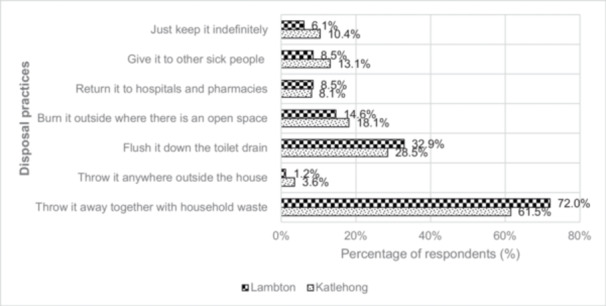
Comparison of HPW disposal practices between study areas.

Disposing of unwanted or expired medicines together with household waste or flushing them down toilets remained the dominant practice among households in this study. Disposing unwanted or expired pharmaceutical products together with household waste was reported by 61.5% of respondents from Katlehong compared with 72.0% of Lambton households (Figure 6). Flushing medicines in the toilet drain was reported by 28.5% of respondents from Katlehong and 32.9% from Lambton. Burning medicines outside of households was practiced by 18.1% of Katlehong households and 14.6% of Lambton households. Returning unused medicines to hospitals or pharmacies was rare in both areas, with only 8.1% in Katlehong and 8.5% in Lambton reporting this practice. *χ* ^2^ analysis showed that there were no statistically significant differences in disposal practices between Katlehong and Lambton (*p* > 0.05), thus indicating that unsafe disposal practices are common across both communities, regardless of socio‐economic differences. These patterns demonstrate that unsafe disposal is prevalent across both communities, regardless of socio‐demographic discrepancies. This may be attributed to shared challenges such as a lack of awareness, absence of adequate disposal facilities, and limited guidance from healthcare professionals.

These findings are consistent with results stemming from community‐based household studies conducted in urban and peri‐urban settings in countries such as Nigeria, India, and Kenya, where disposal of medicines as part of household waste and flushing them into sewer systems were identified as dominant malpractices due to limited access to formal take‐back systems and inadequate public awareness [2, 6, 35, 36].

#### 3.1.6 Reasons for Improper Disposal of HPW

**Figure 7:**
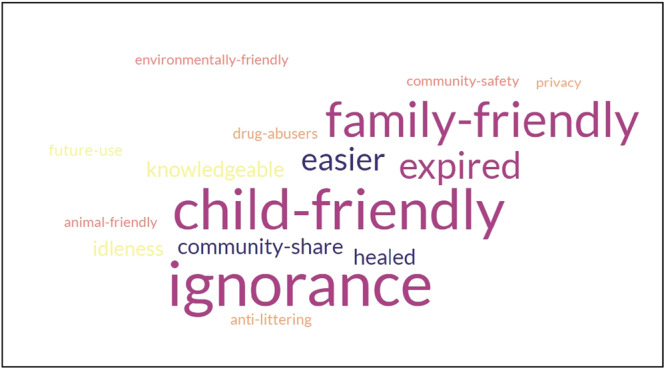
Word frequency chart representation of respondents' perceptions on pharmaceutical waste and medicine disposal.

As shown in Figure 7, child and family safety emerged as dominant drivers of HPW disposal patterns across the sample. To this extent, respondents expressed their experiences as follows:

- “To avoid children or adults taking them by mistake, thinking they are sweets”; and
- “I burn medical waste because I don't want children to get hold of such products.”

A second strong theme that emerged from the analysis was the lack of guidance, this being reflected in admissions such as “I was never instructed or advised on how to dispose of them”, thus indicating limited professional counseling. Convenience and social norms also shaped waste management practices, with households describing disposal as “it is the simplest way to dispose them” or mentioning that “it's normal in my community to share medicine among each other for common sicknesses like flu”. Many respondents (76.3%) therefore discarded medicines as part of household waste or gave them to others because these were familiar, customary practices. A smaller group of respondents (25.5%) expressed environmental or personal safety concerns, as they used terms such as “environmentally friendly disposal of wastes”, “anti‐littering”, and “privacy”. Similar drivers of improper pharmaceutical disposal have been reported in community‐based studies in low‐ and middle‐income countries, where lack of awareness, convenience, and absence of formal disposal systems are consistently identified as key influencing factors [6, 37]. These reasons were observed across both Katlehong and Lambton, with no major differences between the two areas, thereby suggesting that factors such as convenience, lack of guidance, and safety concerns are shared across different socio‐economic settings.

### 3.2 Influence of Demographic and Economic Factors on HPW Storage

The relationship between demographic and economic factors on household pharmaceutical storage practices was also assessed using *χ* ^2^ tests. As shown in Table 4, the analysis revealed no statistically significant associations between safe storage practices and gender (*χ* ^2^ (2, 303) = 0.788, *p* = 0.674), age (*χ* ^2^ (5, 303) = 2.198, *p* = 0.821), employment status (*χ* ^2^ (5, 303) = 1.989, *p* = 0.851), education level (*χ* ^2^ (6, 303) = 8.480, *p* = 0.205), or household income (*χ* ^2^ (5, 303) = 2.566, *p* = 0.767). These findings suggest that safe medicine storage practices were relatively consistent across these demographic groups. However, a statistically significant association was observed between place of residence and safe storage practices (*χ* ^2^ (1, 303) = 4.466, *p* = 0.035). This indicates that the location in which participants resided significantly influenced how medicines were stored in their homes.

**Table 4:** Association between safe storage practices and demographic variables.

Variable	*χ* ^2^ (df)	*p* value
Gender	0.788 (2)	0.674
Age	2.198 (5)	0.821
Employment status	1.989 (5)	0.851
Education level	8.480 (6)	0.205
Household income	2.566 (5)	0.767
Place (suburb)	4.466 (1)	0.035

**Figure 8:**
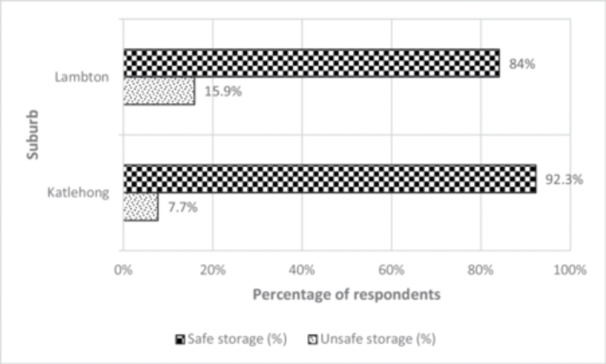
Safe storage practices by place of residence.

Further examination of the distribution revealed that participants residing in Katlehong exhibited higher safe storage practices (92.3%) compared to those residing in Lambton (84.1%) (Figure 8).

#### 3.2.1 Magnitude and Direction of the Cause‐and‐Effect Relationship Between Place of Residence and Safe Storage Practices

The strength of this association was found to be small and positive, as indicated by the Phi coefficient (*Φ *= 0.121). Although the effect size is small, the statistically significant result suggests that the place of residence significantly influenced household medicine storage behavior. This is consistent with previous findings that local places of living and community health exposure shape household medicine‑handling practices [38, 39]. The Relative Risk (RR) further indicated that participants residing in Katlehong were 2× less likely to report unsafe storage practices compared to those in Lambton (RR_lower socioeconomical class suburb_ = 0.442, 95% CI: 0.204–0.957). This may be because commonly crowded living situations within lower socioeconomic suburbs may drive them to be cautious of where they store their pharmaceutical products.

#### 3.2.2 Comparison of the Different Medicine Storage Practices Used in Katlehong and Lambton

**Figure 9:**
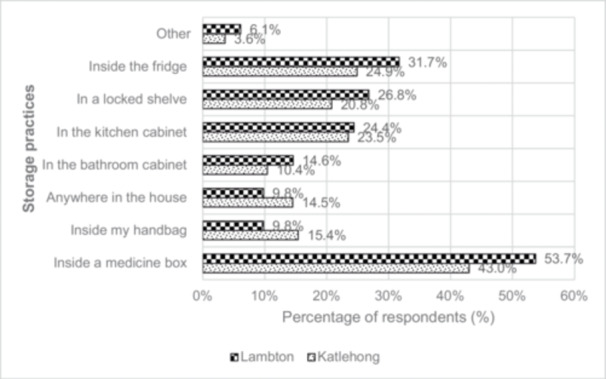
Comparison of household medicine storage practices between Katlehong and Lambton respondents.

While storing medicines inside a medicine box was the most common method used in the survey (45.9%, *n * = 139), there were varied household approaches to medicine storage, with some clear preferences emerging across the two study areas. Notably, 43.0% of respondents in Katlehong (*n *= 95 of 221) and a slightly higher 53.7% in Lambton (*n *= 44 of 82) used this practice (Figure 9). Kitchen cabinets were used by nearly similar proportions in Lambton (24.4%) and Katlehong (23.5%), while locked shelves accounted for 26.8% in Lambton and 20.8% in Katlehong. Domestic refrigerators were also somewhat more frequent in Lambton (31.7%) than in Katlehong (24.9%). Less secure storage areas, such as handbags, bathroom cabinets, and open household spaces, were reported more often in Katlehong. These patterns indicate that Lambton households more often used enclosed or temperature‐controlled spaces, whereas Katlehong respondents reported higher use of open and easily accessible locations. This difference may be attributed to variations in household conditions, storage space availability, and levels of awareness regarding safe medicine storage practices between the two areas. Such patterns correspond with findings from community‐based household studies conducted in urban and peri‐urban settings in Nigeria and Kenya, which similarly reported that, despite awareness of safe‐storage guidelines, medicines were often kept in moisture‐prone or easily accessible locations [32, 40, 41].

**Figure 10:**
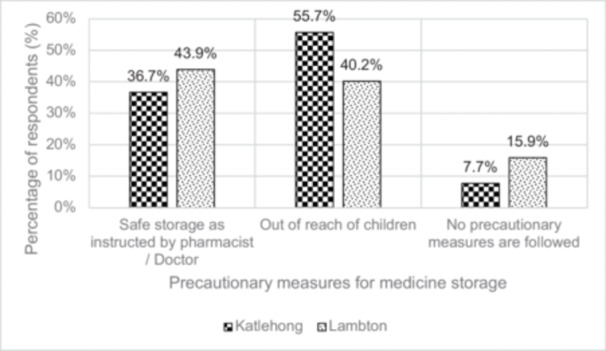
Comparison of precautionary measures for medicine storage between Katlehong and Lambton households.

Across both study areas, the most common precaution was keeping medicines out of reach of children, as it was reported by 51.5% (*n *= 156 out of 303 respondents) of all households (Katlehong 55.7%, Lambton 40.2%) (Figure 10). Overall, 38.6% (*n* = 117) of participants reported following storage instructions provided by pharmacists or doctors, thus making it the second most common precaution (Katlehong: 36.7%; Lambton: 43.9%). Notably, 9.9% of respondents admitted to taking no precautionary measures, with this practice more prevalent in Lambton (15.9%) than Katlehong (7.7%). These findings indicate that, although child safety was considered by many households, gaps remain in the consistent adherence to professional storage guidance, a trend also reported in similar studies [3, 11, 42]. This may reflect limited interaction with healthcare professionals or inconsistent communication of storage guidance at the household level.

#### 3.2.3 Household Willingness to Participate in Pharmacy‐Based Medicine Disposal Programs

**Figure 11:**
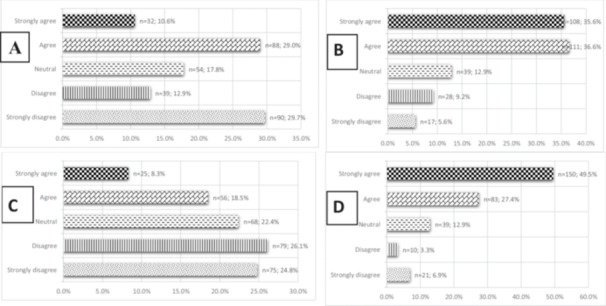
Different levels of agreement/disagreement with the statement “I would return the unused medicine to the pharmacy” (A), “I would highly recommend the program to other people” (B), “I intend to continue using the same disposal method rather than traveling to dispose of the unused medicines or nutraceuticals” (C), and “I intend to follow the advice given to me by health providers” (D).

Overall, the findings suggest that households in Katlehong and Lambton are generally willing to adopt safer pharmaceutical disposal practices, especially if such programs are recommended to the patients by healthcare providers. In both Katlehong (60.6%, *n =* 134) and Lambton (57.3%, *n =* 47), many households expressed varying degrees of willingness to participate in pharmacy‐based medicine disposal programs. While willingness to recommend the programs is high, practical barriers such as travel and convenience could limit their adoption if the necessary infrastructure is not made accessible for HPW collection. Nearly four in ten respondents (39.6%) agreed (agree or strongly agree) with returning unused medicines to the pharmacy, while a significant proportion (42.6%) either disagreed or strongly disagreed (Figure 11).

#### 3.2.4 Recommending Medicine Waste Take‐Back Program to Others

The majority (72.2%) of respondents indicated that they were willing to recommend the disposal program to others, with 36.6% agreeing and 35.6% strongly agreeing (Figure 11B). Only a small group of respondents (14.8%) opposed the idea, while 12.9% were neutral. When disaggregated by study area, both Katlehong and Lambton respondents showed similarly high levels of agreement, thus indicating strong support for recommending such programs across both communities (*p* < 0.05). This demonstrates clear community support and potential for widespread acceptance. In this manner, these findings align with the results expressed by Hiew and Low [1], who found high levels of positive attitudes among healthcare professionals and students towards safe pharmaceutical waste disposal initiatives, thus indicating similar willingness to support such programs.

#### 3.2.5 Continuing Current Disposal Methods Rather Than Traveling to Dispose of the Unused Medicines or Nutraceuticals

Just over half of respondents (50.9%) rejected the idea of sticking to their current disposal practices if safer alternatives required traveling, while 26.8% indicated they might continue (Figure 11C). About 22.4% remained neutral. Both areas indicated reluctance to travel any distance for disposal purposes despite willingness to adopt safer practices (*p* > 0.05). This suggests a clear contrast between willingness to adopt safer practices and the need to overcome practical barriers, such as accessibility and convenience, across both socio‐economic contexts. In a related study, Michael et al. [43] reported similar concerns in Nigeria, where logistical barriers limited household participation in safe medicine disposal schemes despite relatively high levels of awareness. Similar patterns have been reported in community‐based studies in countries such as Sri Lanka, where households expressed willingness to participate but were constrained by the absence of proper disposal guidance and well‐organized waste management systems [10].

#### 3.2.6 Following Advice From Health Providers

The majority (76.9%) of respondents (agreed and strongly agreed) indicated they would follow the advice of healthcare providers on safe medicine disposal. Only 10.2% of respondents disagreed, while 12.9% were neutral (Figure 11D). This shows that health professionals remain a trusted source of authority in influencing household practices around HPW management across both communities. Consumers expressed willingness to adopt safer disposal methods when recommendations came from healthcare professionals, thus emphasizing the crucial role of trusted advice [44, 45, 46].

## 4 Strengths and Limitations of the Study

This study provides important insights into HPW management practices across two socio‐economically contrasting communities within the same municipality. A key strength of the study is its comparative design, which allowed for the identification of similarities and differences between a township and a suburban setting. In addition, the use of a mixed‐method survey approach, which has incorporated both quantitative and qualitative data, has enabled a better understanding of HPW management practices, perceptions, and underlying reasons for associated patterns.

Although the research aim was achieved, a few limitations were encountered. Firstly, the scope was restricted to only two communities (Katlehong and Lambton), which limits the extent to which the findings can be generalized to other parts of South Africa with differing socio‐economic, cultural, or infrastructural conditions. Secondly, access to respondents posed challenges, as some households were reluctant to participate, thereby potentially affecting the diversity of responses. Thirdly, the study relied on self‐reported data, which carries the risk of recall bias and the influence of social desirability on responses. Finally, the lack of formal pharmaceutical waste disposal facilities, such as take‐back systems, in South Africa restricted opportunities to compare proper and improper disposal methods more systematically.

### 4.1 Conclusions and Recommendations

Given the adverse environmental and health impacts of poorly managed HPW globally, the current research sought to bring a better understanding of different types of items in this waste stream at the household level, their storage and disposal practices, as well as the willingness of households to participate in more sustainable waste management practices in the EMM of Gauteng Province, South Africa. More specifically, a mixed‐method survey was undertaken in two socio‐economically contrasting study areas, Katlehong Township and Lambton Suburb, to demonstrate similarities and discrepancies and to suggest implications for more sustainable domestic and household waste management practices. The results are summarized in this section along with recommendations to overcome challenges and constraints in the storage and disposal of HPWs.

#### 4.1.1 Summary of Findings and Their Synthesis

The study revealed that HPW is a significant public health and environmental concern in the selected study areas, particularly within the contrasting socio‐economic contexts of Katlehong Township and Lambton Suburb. However, awareness of this waste stream was generally low, with only 40.3% of respondents indicating knowledge of HPW. This reflects the broader challenge of inadequate public education on HPW management. The role of healthcare professionals in guiding disposal was found to be limited, since most respondents (71,6%) never received advice from healthcare professionals on medicine disposal. The comparative analysis (*χ* ^2^ tests) between Katlehong and Lambton showed that household income (*χ* ^2^ (1, 303) = 20.989, *p* < 0.001, *Φ* = 0.118, RR_lower income_ = 0.245, 95% CI: 0.934–24.341) is a significant economic factor that explained HPW disposal behavior in the study areas. Child and family safety emerged as dominant drivers of HPW disposal patterns across the sample. Moreso, the suburb where a respondent resided could explain the pharmaceutical storage practices, with safer storage being seen in Kahlehong (*χ* ^2^ (1, 303) = 4.466, *p* = 0.035, *Φ* = 0.121, RR_lower socioeconomical class suburb_ = 0.442, 95% CI: 0.204–0.957). However, the rest of the demographic (age, gender) and economic (educational level, household income) factors tested in this study could not explain the patterns reflected, thus indicating that demographic or economic context alone does not predict safer household pharmaceutical storage and waste management in this study. At the same time, both communities shared common challenges such as limited awareness, the absence of disposal facilities, and the lack of advice from healthcare professionals. Overall, the study highlights the need for appropriate interventions, including community awareness campaigns, pharmacy‐led take‐back programs, and stronger integration of HPW management into municipal waste planning.

#### 4.1.2 Summary of Recommendations and Implications

Based on the above conclusions, several recommendations are proposed:

- Government, municipalities, and NGOs should prioritize public education campaigns on HPW, which emphasize environmental and health risks, proper storage, and safe disposal options for pharmaceutical products. Community workshops, media platforms, and health clinics can be leveraged to spread the message.
- Pharmacists and healthcare workers should be trained and encouraged to routinely advise patients on safe disposal, as recommended by Chandrasena et al. [10]. This requires integrating HPW education into healthcare service delivery so that patients receive appropriate guidance.
- Alongside education, accessible medicine take‐back programs should be established at pharmacies and health facilities. Models from high‐income countries show that such schemes provide safe alternatives and significantly reduce reliance on unsafe household practices (WHO, 2014).
- At the policy level, the National Environmental Management: Waste Act (Act No. 59 of 2008) already provides a sound framework for the development of Integrated Waste Management Plans, yet enforcement remains limited. Municipalities should therefore integrate HPW into their IWMPs and establish local disposal infrastructure.
- Finally, further research should extend beyond the two study areas selected in the current study, the goal being to investigate other socio‐economic contexts. Such future studies can include longitudinal assessments to track behavioral changes over time. Studies like these can strengthen the evidence base and guide tailored interventions, thus allowing for comparative analyses at a national scale.

## Author Contributions

**Sithembile Mduduzi Tshomela:** conceptualization, methodology, investigation, writing – original draft, writing – review and editing, visualization, formal analysis, project administration, data curation, funding acquisition. **Isaac Tebogo Rampedi:** conceptualization, writing – review and editing, supervision, resources, funding acquisition, validation, methodology, writing – original draft, project administration. **Phyllis Rumbidzai Kwenda:** conceptualization, methodology, investigation, writing – review and editing, visualization, formal analysis, supervision, project administration.

## Ethics Statement

The research was approved by the University of Johannesburg (Ethics Reference Number: 2025‐07‐10/Tshomela_Rampedi).

## Consent

The study obtained prior informed consent by explaining its objectives, procedures, risks, and respondents' right to withdraw at any time.

## Conflicts of Interest

The authors declare no conflicts of interest.

## Transparency Statement

The corresponding author, Phyllis R. Kwenda, affirms that this manuscript is an honest, accurate, and transparent account of the study being reported; that no important aspects of the study have been omitted; and that any discrepancies from the study as planned (and, if relevant, registered) have been explained.

## Data Availability

The data that support the findings of this study are available from the corresponding author upon reasonable request.
